# Progress in Surgery

**Published:** 1895-04-27

**Authors:** 


### Progress in Surgery.

### SURGERY OF THE INTESTINES.

(*Continued from page* 120, *Vol. XVII.*)

**Laparotomy for Perforating Ulcer of the Intestines** continues to receive a large share of attention. Marmaduke Sheild,[1] in speaking of duodenal ulcers, formed the following conclusions: (1) That perforating ulcer of the duodenum was a rare affection, and far more common in young adult males than females. (2) That in a considerable number of cases the symptoms were primarily epigastric or referred to the right hypochondrium, or there was a previous history of epigastric troubles. (3) That in perforative peritonitis occurring in a male, if the symptoms were not very typical of implication of the vermiform appendix, the surgeon should turn his attention to the duodenum, where the lesion would most generally be found. The absence of fæcal gas or odour in the abdominal contents pointed to implication of the duodenum or stomach. Acidity of the abdominal contents pointed to the same conclusion. A small exploratory incision below the umbilicus would clear up the nature of the abdominal contents. (4) The success of the future lay in the cleansing the peritoneum by repeated hot water flushings and drainage of the pelvic cul-de-sac. Abbe[2] has collected seventeen cases of operation for perforating typhoid ulcers, and of these three recovered.

**Intestinal Approximation by Means of Murphy's Button** has been the subject of experiment by Chaput,[3] who says there is no question that this ingenious instrument greatly shortens the duration of intestinal operations, renders them easier, and prevents obstruction, but unfortunately it is not free from danger, and its application requires great prudence and skill. In a series of 24 experiments on dead bodies, in respect of passing Murphy's buttons through the intestines, he obtained the following results from the use of the smallest size: Of the 24 cases this button was completely stopped in 3; it passed with great difficulty in 6, the obstacle being only overcome by extreme insufflation and energetic pressure with the fingers; in 15 it passed through readily. Murphy[4] himself says that the button has now been used in a sufficient number of cases to enable us to make valuable deductions for our guidance in the future. His general conclusions are as follows: 1. The cicatrix produced with the button does not contract. 2. In intestinal obstruction resection with end-to-end union gives better results than lateral approximation, and should always be performed where practicable. The same operation should always be done in gangrenous hernia. In fæcal fistula the bowel should be resected and united end to end. 3. The patients should receive liquid nourishment as soon as the effect of the anæsthetic passes off. The bowels should be made to move as soon as possible after the operation, and frequent evacuations kept up. 4. If the button does not pass in three or four weeks the rectum should be examined, as it may rest just inside the sphincter. 5. There has been one case reported of occlusion of the button by fæcal impaction in the cylinder. This can be easily avoided by mild cathartics immediately after operation. 6. When returning to the abdomen the intestines should be placed in parallel lines, especially at the seat of approximation, to prevent sharp curves and obstructions. This occurred once with the button; many are reported following suture, 7. There is no danger from obstruction from the button, as not a single case has been reported. This proves that the deductions made by Chaput, of Paris, from experiments on the cadaver are erroneous. 8. There is no danger of extension of the pressure atrophy beyond the line of pressure. 9. Primary adhesion may be hastened in malignant cases by abrading the peritoneum with a needle. It is unnecessary in non-malignant cases. 10. A supporting suture is never necessary to secure union, and should only be used to relieve tension when the viscera approximated are forced out of position. 11. The mucous membrane should be pushed down in the cup of the button before closing it; if redundant it should be trimmed off with scissors. It should never be allowed to protrude between the edges of the button when the button is closed. 12. The following points regarding the construction of the button should be noted before using it: (*a*) The spring catches should hold firmly in all positions and should be made of a metal that will not be corroded by acids. (*b*) The elastic pressure cup should be on the male half of the button (never on the female). (*c*) The edges of the pressure surface should be very smooth and hemispherical in shape. (*d*) The spring under the pressure cup should not be too strong. 13. If the button appears at the opening of the fistula after lateral approximation, do not try to force it through the opening, it is unsurgical; open the abdomen and (*a*) press it back to the anastomotic opening and through it onwards down the intestine and it will pass, or (*b*) make a longitudinal incision in the bowel and take it out. Villard[5] concludes from experimental researches on dogs that Murphy's method is bound to take an important place; the simplicity of the operative proceedings and the rapidity of execution, with the perfection of the co-aptation, must tend to make its use general.

**The use of absorbable plates in visceral approximation** has been the subject of investigation by Magill,[6] who finds that the general mortality of 87 operations in which they were used was 23·10 per cent. Of these 87 cases, 61 were gastro-enterostomies with a mortality of 22·95 per cent.; 3 pylorectomies with a mortality of 33·33 per cent.; 2 cholecystenterostomies, with no mortality; and 21 were cases of anastomosis of intestine only, with a mortality of 23·80 per cent. The plates have generally been absorbed, sometimes eliminated in fragments, never having caused any trouble. Magill concludes as follows: The superiority of visceral approximation with absorbable plates is clinically established when compared with methods of suture, and should cause their rejection. The rapidity of plate approximation makes pylorectomy a different operation to what it was when it was performed with sutures. Its possibilities are therefore greater, and should make surgeons prefer pylorectomy rather than gastro enterostomy. The brilliant results of Senn's method have changed the indications of treatment of visceral stenosis. The safety of operation is assured. Baracz[7] prefers the use of prepared vegetable (turnip) plates, of the same shape and size as the ordinary Senn's plates, as they never produce necrosis and perforation, as has occurred with bone plates not sufficiently decalcified.

**Intestinal Obstruction.**—Obalinski[8] discusses diagnosis and treatment on the ground of 110 cases in which he has performed laparotomy for this condition. Out of this number he claims 38 successes, or 34·5 per cent. The local signs to which he directs attention, as distinguishing mechanical from paralytic obstruction are (1) increased peristalsis and (2) local meteorism. The former is absent in the acute obstruction of external hernia, and both are absent in the more advanced stage, in the presence of general peritonitis and paralysis of the intestine, with general tympanites. His conclusion is that where we find, in addition to the ordinary symptoms of intestinal obstruction, such as constipation, vomiting, pains, and distension of the abdomen, also one of these characteristic features, namely, increased intestinal movement and local meteorism, we are in a position to diagnose a mechanical obstruction which can only be radically relieved by an operation. Both symptoms may be present in a case where the segment of bowel involved is not so small as to disappear among the other coils; on the other hand, the affected segment must not be so large as to obscure the movements of the section of bowel higher up. Increased peristaltic movement will appear alone when the strangulated loop is very small or lies deep in the abdomen, or when the mechanical obstruction is due to a simple transverse constriction (by band), or to plugging by a stone, tumour, or the like. Local meteorism will appear alone where the strangulated piece of bowel is very large, as in rotation of a large portion of the small intestine or sigmoid flexure. In presence of a general ballooning of the abdomen without obvious increased peristalsis, and without demarcation of individual coils we shall diagnose functional obstruction (ileus paralyticus), which may have resulted either from escape of the contents of one of the viscera into the peritoneal cavity, or from acute inflammation of one of these viscera attended by intra-peritoneal or sub-peritoneal exudations. Eve[9] has collected twenty-seven cases of laparotomy for intestinal obstruction due to gall stones. Of these only nine recovered. He made the following suggestions with regard to treatment in such cases:—(1) When a diagnosis was possible expectant treatment should be tried, as one-third of the cases of marked obstruction from gall stones recovered without operation. (2) But operative treatment should not be long delayed, as the patients were nearly all aged, the mean age being sixty-four years. (3) After opening the abdomen, the calculus should, if possible, be forced through the ileo-cæcal valve. (4) If the calculus be immovable, or at a distance from the valve, and the intestine healthy, the stone should be excised. (5) When the intestine was inflamed, and the stone immovable and adherent to it, an attempt might be made to break the calculus up with needles. This failing, it should be extracted, the intestine fixed to the abdominal wall near the wound, and surrounded with iodoform gauze. (6) In only one case out of twenty-seven was it found necessary to resect the intestine on account of ulceration.

Elliott[10] reports a case of strangulation of Meckel's diverticulum, caused by volvulus of the ileum, which was supposed to be a case of appendicitis. The case is important, because it impresses the fact that strangulation of Meckel's diverticulum is an occasional cause of acute peritonitis, and because the diverticulum somewhat resembles the vermiform appendix, the former in some cases even having a mesentery. The symptoms of affections of the two structures would be much the same. The presence of a tumour, and marked tenderness near the umbilicus, and the history of a discharge from the umbilicus, are both significant of the presence of the diverticulum. In either affection there is apt to be a history of previous attacks of pain.

[1] Lancet, Oct. 27th, 1894, p. 978. [2] Med. Record, New York, Jan. 5th, 1895, p. 1. [3] Med. Week., Nov. 9, 1894. [4] Chicago Clinic. Review, Feb., 1895, p. 248. [5] Med. Chron., Dec., 1894, and Lyon Med., Oct. 7th, 1894. [6] Annals of Surgery, Sept., 1894, p. 297. [7] Amer. Journ. of the Med. Sci., Nov., 1894, p. 599. [8] Glasgow Med. Journ., Sept., 1894, and Arch. f. Klin. Chir., Bd. 48, Hft. 1. [9] Lancet, Jan 19th, 1895, p. 156. [10] Amer. Journ. of the Med. Sci., Nov., 1894, p. 605.

